# Adam a Nation

**Published:** 1881-04

**Authors:** W. H. Ruff

**Affiliations:** Glympville, South Carolina


ARTICLE III.

### ADAM A NATION.

BY W. H. RUFF, M. D., GLYMPVILLE, SOUTH CAROLINA.

In the INDEPENDENT PRACTITIONER, Vol. II, January, 1881, No. 1, Article I, headed "Preadamite Races of Men," the author says: "The subject presents the deepest interest." To this we say, It does; and we will set forth a few facts, showing Adam was a Nation, *not* a Man. The so-called flood has led the world astray on this point. Flood is a *trope*, and so is fire used to show the impossibility of escaping the sentence of God, when it has passed; as, "Pray not for this people, for their good."—Jer. xiv ch., 11. "The prophet's prophecy lies in my name," 14. Jesus said: "All the shepherds that ever came before him were thieves and robbers."—John, x ch., 8, see the 1st verse. And Jer. xiv ch., 14th and 15th: "By sword and famine shall those prophets be consumed;" 15th, "Cast them out of my sight, and let them go forth."—Jer., xv ch., 2d: "And if they say unto thee: whither shall we go forth? then thou shalt tell them: Thus saith the Lord; such as are for death, to death; such as are for the sword, to the sword; such as are for famine, to the famine; and such as for the captivity, to the captivity, 2. And I will cause them to be removed into all kingdoms of the earth, 4. "Behold, every one that useth proverbs, shall use this proverb against thee, saying: as is the mother, so is her daughter."—Ezekiel, xvi ch. 44th v.

The laws of God changeth not; therefore the same penalties that were for Adam, were for Israel; and the same covenant that was made for Israel, was for Adam and his mother, ages upon ages untold; every rule in every science, is the law of God; as twice two is four; it has never changed, and never will.

*White parents will breed white children*, and *black will breed black children.* This is known by a test of five thousand years. *Like will produce like*; a turkey hen will lay as many eggs without a gobbler, as with one; but the eggs will not hatch; without copulation any race will cease to exist; and to *mingle the races of man, will adulterate both, or all the races that do mingle*; and *this* was what brought upon the sons and daughters of Adam, who were also the sons and daughters of God, *death*, *sword*, *famine and captivity*. And *Adam the nation*, was cast out of the Garden of God, the garden of Eden, into Nod, as Israel, the ten tribes of Israel were cast out into Assyria, for eating things offered to Baal, and they were put under task-masters, to get their bread by the sweat of their brow, as Adam was in the land of Nod, and as Israel was in Egypt, and all who were cast out of the garden of God should return *no* more, nor see their native country. But their children might, by complying with the terms of the covenant, which was made with Abraham.—Lev. xxvi, 40–41. The *mingled* people were destroyed. See Ezekiel xxx, 5, Ezra ix. The flood is used as a trope, and could never have been water, unless the law was changed.

The King of Tyrus was in Eden.—Ezekiel xxviii ch. 13. And he was *perfect* in *his* ways, until *iniquity* was found in him, 15.

The Assyrian was a cedar in Lebanon, with fair branches, &c. Ez. xxxi ch., 3. The cedars in the garden of God could not hide him, 8. The *trees* that were in the garden of God, envied him. Ezekiel xxxi ch., 9. And so they did Adam and her daughter Israel.

It is the *mis* use of words that has led to such awful mistakes. Thus saith the law: "The father shall not be put to death for the sins of his son; nor the son for the sins of his father. As I live, saith the law, the soul that sinneth, *it* shall die."

Both Moses and Jesus said: "No man ever heard the voice of God," yet this voice is heard in all His laws, and in *no* other way; as by *fire* and by *sword* will the Lord plead with all flesh. This is the voice of God, and known to be true; and the result being known, it has become an axiom, and can be made no plainer by demonstration. If you give emetics you expect to empty the stomach, &c. And you may say: "Thus saith the Lord;" or, "thus saith the law." It is the *law* that foretells, and *not man*. And when any result is found in any of the sciences, it then becomes an axiom, or a known law of God, and can be relied upon as true. The Bible is a *forgery* and *Christianity* as it is *now* taught, is a *fraud*. The laws of God are *now* what they have *always* been. Jesus taught the same laws that Moses taught. Being asked by a lawyer: "What was the first commandment?" He said: "Hear, O Israel! the Lord our God is one Lord." And then gave the commandments as Moses did. Jesus was a *saviour*, *not* a *redeemer;* for redeem implies that *some* being had became *greater*

than *God.* This is the false notion of the heathen. They taught there were many gods, and these false notions have been incorporated into the laws of Moses, which are also the laws of God. They never in any age demanded burnt offerings and sacrifices of any kind—see Isaiah, i ch. Paul says, "*without* the shedding of blood there is no remissions." You do not bleed the father instead of the son, who needs the bleeding; and so the *soul*, that is the *man* or *nation* that *needs* bleeding, is *bled*, and if *no* repentance, *he* or *it* is *bled again* and *again*, and *if* the *disease*, to use a trope, *yields not*, *then captivity* follows. This is the law for all nations.

Jacob, who was also called Israel, was cast out of Eden into Egypt for idolatry. And when it be asked why the Lord God did it, It shall be answered: "Because he forsook the Lord God and broke his covenant, which he made with their fathers when he brought them out of Egypt."—Deut. xxix ch., 24–25. See also II. Chron. vii ch., 21–27, also Jer. v ch., 19, and Jer. xi ch., 1–7: Saying, "Obey, my voice," but they obeyed not, 7.

The covenant referred to, see Exodus xix ch., 3-6. The terms are: "If ye will obey *my voice* indeed, and keep *my* covenant, *then* ye shall be a *peculiar* treasure unto *me* above *all* people; for *all* the *earth* is *mine;* and ye shall be unto me a kingdom of Rulers, not Priests, as in the text, and an holy nation.

Now, when Joshua led them into their inheritance, and divided the land among them they were called Israel, and they were a kingdom of rulers, and an holy nation, according to the terms of the covenant. And so was Adam the nation called Adam when they came back into the kingdom of God. And when they broke the covenant, *they* or *he* was cast out, and sent back to till the land from which they had been taken, and their name was called Adam.—Gen. v, 2. All the pronouns are used in speaking of them as if it was a man. Edom, Moab and Ammon are spoken of to the days of Jeremiah and Ezekiel as a man.

And the Lord said unto Moses, in Midian: "Go, return into Egypt, for all the men are dead which sought thy life. Ex. iv, 19. And thou shall say unto Pharoah: Thus saith the Lord: Israel is my son, even my first born, 22. Let *my* son go, and *if* thou *refuse* to let *my* son go, that he may serve me; behold I will slay *thy* son, even *thy first* born, 23. Both were nations. For want of space we close.

